# The CD36 Ligand-Promoted Autophagy Protects Retinal Pigment Epithelial Cells from Oxidative Stress

**DOI:** 10.1155/2021/6691402

**Published:** 2021-03-28

**Authors:** Marie-France Dorion, Mukandila Mulumba, Shuya Kasai, Ken Itoh, William D. Lubell, Huy Ong

**Affiliations:** ^1^Department of Neurology and Neurosurgery, Montreal Neurological Institute, McGill University, 3801 University Street, Montreal, QC, Canada H3A 2B4; ^2^Faculty of Pharmacy, Université de Montréal, 2940 Chemin de Polytechnique, Montreal, QC, Canada H3T 1J4; ^3^Department of Stress Response Science, Center for Advanced Medical Research, Hirosaki University Graduate School of Medicine, 5 Zaifu-cho, Hirosaki 036-8562, Japan; ^4^Department of Chemistry, Université de Montréal, 2900 Boulevard Édouard Montpetit, Montreal, QC, Canada H3T 1J4

## Abstract

The retinal pigment epithelium (RPE) performs many functions that maintain photoreceptor health. Oxidative damage to the RPE is a critical component in the pathogenesis of eye diseases such as age-related macular degeneration (AMD). Ligands of the cluster of differentiation 36 (CD36) have previously preserved photoreceptor integrity in mouse models of AMD. The cytoprotective effect of the CD36 ligand MPE-001 on RPE cells has now been elucidated employing a model of oxidative stress. Sodium iodate (NaIO_3_) induced formation of reactive oxygen species and apoptosis in human RPE cells, which were decreased by MPE-001 without affecting antioxidant enzyme transcription. Immunoblotting and immunostaining assays showed a restorative effect of MPE-001 on the autophagic flux disrupted by NaIO_3_, which was associated with an increase in syntaxin 17-positive mature autophagosomes. The cytoprotective effect of MPE-001 was completely abolished by the autophagy inhibitors wortmannin and bafilomycin A1. In conclusion, we report for the first time an autophagy-dependent protection of RPE cells from oxidative stress by a CD36 ligand.

## 1. Introduction

The retinal pigment epithelium (RPE) is a monolayer of polarized epithelial cells located posterior to the neuroretina in close contact with photoreceptors. Responsible for the maintenance of photoreceptors, the RPE performs daily phagocytosis of shed photoreceptor outer segments (POS). High metabolic activity combined with exposure to light and photochemical reactions in the oxygenated environment render the RPE prone to oxidative stress. Oxidative damage to the RPE can lead to dysfunction with cell loss, inflammation, and degeneration of photoreceptors, characteristics of age-related macular degeneration (AMD) [1].

AMD is a leading cause of visual impairment in the elderly population [2] and is categorized into wet (neovascular) and dry (atrophic, nonneovascular) forms with distinct pathophysiological features. Hallmarks of dry AMD include RPE abnormality with accumulation of a complex deposit of lipids and proteins called drusen and atrophy of the photoreceptor layer in the macular region of the retina [1]. Although therapy is available for the wet form, no cure nor treatment exists to prevent dry AMD [3]. The role of oxidative stress in the pathogenesis of AMD is supported by observations of an AMD-like phenotype in mice with a genetic deficiency in either superoxide dismutase 1 or 2 (*Sod1* [4] or *Sod2* [5]) or nuclear factor erythroid 2-like 2 (*Nfe2l2*) [6]. Furthermore, smoking tabacco is a risk factor of AMD [7]. Increased expression of antioxidant enzymes [8] and mitochondrial DNA damage [9] have been observed in the eyes of AMD patients.

Autophagy is an adaptive mechanism for recycling intracellular components to protect cells against oxidative damage. During autophagy, specialized double-membrane vesicles called autophagosomes form, targeting and engulfing damaged proteins and organelles for lysosomal degradation. After autophagy initiation, the nascent double-membrane phagophores elongate to sequestrate cargo and close to form mature autophagosomes, which subsequently fuse with lysosomes [10]. Autophagy malfunction has been associated with the pathogenesis of AMD. Decreased autophagic degradation activity, the so-called autophagic flux, and increased susceptibility to oxidative damage have been observed in the RPE cells of AMD patients [11]. Moreover, RPE-specific deficiency of autophagy-related 5 or 7 (*Atg5* or *Atg7*) genes has been shown to lead to retinal degeneration in mice [12].

The scavenger receptor cluster of differentiation 36 (CD36) is well known as a transporter of fatty acids into cells [13]. Playing a role in the innate immune response and lipid homeostasis, CD36 is expressed in diverse cellular types including RPE cells [14]. Endogenous ligands of CD36, several of which promote inflammation, are known to accumulate within the retina in AMD, including *β*-amyloids [15], oxidized low-density lipoproteins [16], and advanced glycation end products [17]. In addition, CD36 is involved in lipid handling in the diseased retina by mediating the clearance of subretinal deposits in mice [18]. The selective CD36 ligand EP80317, a derivative of growth hormone-releasing peptide-6 (GHRP-6), has been shown to preserve photoreceptor function in *Apoe* − /− mice fed a high-fat high-cholesterol diet [18]. Furthermore, the azapeptide MPE-001 (His-D-Trp-Ala-azaTyr-D-Phe-Lys-NH_2_), a semicarbazide derivative of GHRP-6 that exhibits high CD36 binding affinity [19], attenuates photooxidative stress-induced subretinal inflammation in mice and prevents photoreceptor degeneration [20]. However, the effect of these unique synthetic CD36 ligands on RPE cell redox status has yet to be documented.

In the present study, we examined the potential of CD36 as a target to modulate oxidative stress in the RPE. Application of the azapeptide MPE-001 on human RPE cell lines exposed to oxidative stress has elucidated the role of autophagy in the cytoprotective mechanism of action. Oxidative stress was induced by sodium iodate (NaIO_3_), an oxidant known to cause retinal degeneration through selective damage to the RPE in animals [21]. In RPE cell culture systems, NaIO_3_ has been used to reproducibly model oxidative stress involved in AMD development [22–28].

## 2. Materials and Methods

### 2.1. Antibodies and Reagents

Antibodies against HMOX-1 (5061S), GAPDH (2118S), LAMP1 (9091S), and LC3B (2775S) were purchased from Cell Signaling Technology. Antibodies against STX17 (PA5-40127) and ZO-1 (61-7300) were from Invitrogen. Antibodies against TOMM20 (H00009804-M01) were from Abnova. Sodium iodate was purchased from Wako Pure Chemical Industries. Bafilomycin A1, wortmannin, and *N*-acetyl-L-cysteine (NAC) were from Sigma-Aldrich. MitoTEMPO and mitoquinol (MitoQ) were from Cayman Chemical. Trifluoromethoxy carbonyl cyanide phenylhydrazone (FCCP) was from Agilent Technologies. Solutions of NaIO_3_ or NAC were freshly prepared in complete media before each experiment. The pH of NAC-containing media was adjusted back to the original pH using sodium hydroxide.

### 2.2. hTERT RPE-1 Culture

hTERT RPE-1 cells (American Type Cell Collection) were cultured in complete medium (DMEM/Ham's F12 media with 10% FBS, 100 U/mL penicillin and 100 *μ*g/mL streptomycin). Cells were maintained at 37°C under a 5% CO_2_ atmosphere. Cells were used from passages between 8 and 18. After each passage, cells were incubated for at least 48 h to reach 90–100% confluence before treatments, unless otherwise specified.

### 2.3. Reactive Oxygen Species (ROS) Detection

CM-H_2_DCFDA and MitoSOX Red (Invitrogen) were used to assess total cellular ROS and mitochondrial superoxide formation, respectively. Production of ROS was assessed 2 h following NaIO_3_ exposure, before the occurrence of apparent cell death. Cells in a black 96-well plate were incubated with 10 *μ*M CM-H_2_DCFDA for 30 min or with 2 *μ*M MitoSOX Red for 15 min in phosphate-buffered saline (PBS) at 37°C. Fluorescence intensity (excitation/emission = 495/525 for CM-H_2_DCFDA and 510/580 for MitoSOX Red) was measured using a Synergy H1 Hybrid Multi-Mode Microplate Reader (BioTek Instruments). All conditions were analyzed at least in triplicate.

### 2.4. Viability Assay

Cell Counting Kit-8 (CCK-8) (Dojindo Molecular Technologies) was used for the assessment of cellular viability. In a 96-well plate, 2500 cells per well were cultured for 48 h, treated, and assessed for viability following the manufacturer's protocols. All conditions were analyzed at least in triplicate.

### 2.5. Caspase 3/7 Activity

In a black 96-well plate, cells were incubated with 5 *μ*M CellEvent™ Caspase-3/7 Green Detection Reagent (Invitrogen) in PBS with 5% FBS for 30 min at 37°C and counterstained with Hoechst 33342. Image acquisitions and analysis were carried out using a CQ1 confocal quantitative image cytometer to determine the proportion of cells with active caspase 3/7. All conditions were analyzed at least in triplicate.

### 2.6. Mitochondrial Membrane Potential

Mitochondrial depolarization was assessed using JC-1 dye (Invitrogen). In a black 96-well plate, 5000 cells per well were cultured for 48 h, treated, and incubated with 2.5 *μ*g/mL of JC-1 dye in serum-free media for 20 min at 37°C. Fluorescence intensity was measured using a Synergy H1 Hybrid Multi-Mode Microplate Reader, and the red (excitation/emission = 535/590) to green (excitation/emission = 485/530) fluorescence intensity ratio was determined. All conditions were analyzed at least in triplicate. Cells were additionally stained with Hoechst 33342 (1 *μ*g/mL) for imaging purposes. Images were obtained using a ZEISS Axio Observer.

### 2.7. Transfection

For the RNA interference of *CD36*, cells were seeded overnight and then transfected with either ON-TARGETplus human CD36 siRNA SMART pool or siGENOME RISC-Free Control (Dharmacon) using INTERFERin siRNA/miRNA Transfection Reagent (final concentration of 0.15%; Polyplus-transfection). All procedures were performed according to the manufacturer's recommendations. Knockdown efficiency was assessed by qRT-PCR using *GAPDH* and *YWHAZ* as endogenous controls.

For the visualization of autophagosomes and the flow cytometry assessment of autophagic flux, cells were transfected with Premo Autophagy Sensor LC3B-GFP, BacMam 2.0, and a Premo™ Autophagy Tandem Sensor RFP-GFP-LC3B Kit (Invitrogen). Cells were incubated with 40 particles per cell for 48 hours before carrying out downstream assays. All procedures were performed according to the manufacturer's recommendations.

### 2.8. Quantitative Reverse Transcription Polymerase Chain Reaction (qRT-PCR)

RNA was extracted using a RNeasy mini kit (QIAGEN) with some modifications to the recommended protocol. Briefly, cells were lysed with RiboZol RNA Extraction Reagent (VWR Life Science), mixed with one-third volume of chloroform, and centrifuged at 10000 g for 18 min at 4°C. The aqueous phase supernatant was mixed with isopropanol and transferred to RNeasy columns. The manufacturer's protocol was followed from this step onward. The qPCR was performed using a ViiA 7 Real-Time PCR System (Life Technologies). Results were analyzed with the 2^−ΔΔ*Ct*^ method, using *YWHAZ* and *PPIA* as endogenous controls. Primer sequences are shown in supplementary materials (Table S1).

### 2.9. ARPE-19 Cell Differentiation and Treatments

ARPE-19 cells (American Type Cell Collection) were cultured in complete medium and differentiated as previously described with slight modifications [29]. Briefly, cells were seeded at a density of 166000 cells/cm^2^ on Matrigel- (Corning) coated Transwell 96-well inserts (Corning) and cultured for two weeks in MEM-Nic: Minimum Essential Medium alpha with L-glutamine (Wisent Bioproducts), 1% FBS, 100 U/mL penicillin, 100 *μ*g/mL streptomycin, 0.1 mM Non Essential Amino Acids (HyClone), 1% N1 supplement (Sigma-Aldrich), 0.25 mg/mL taurine (Sigma-Aldrich), 20 ng/mL hydrocortisone (Sigma-Aldrich), 0.013 ng/mL triiodothyronine (Sigma-Aldrich), and 10 mM nicotinamide (Sigma-Aldrich). The medium was replaced three times per week. Cells were maintained at 37°C under a 5% CO_2_ atmosphere.

### 2.10. Western Blotting

Cells were washed with ice-cold PBS and lysed with RIPA buffer (150 mM NaCl, 50 mM Tris-HCl pH 7.4, 1% Triton X-100, 0.1% SDS, 25 mM NaF, and 5 mM EDTA with protease and phosphatase inhibitors; Pierce). Cell lysates were centrifuged at 450 g for 30 min at 4°C and supernatants were retrieved. Protein concentrations were determined by the BCA assay (Pierce Biotechnology). Proteins were separated on SDS-polyacrylamide gel and transferred electrophoretically to polyvinylidene difluoride (PVDF) membranes (Bio-Rad Laboratories) for immunoblotting. All primary antibodies were used at a dilution of 1 : 500. Immunoblotted bands were detected by enhanced chemiluminescence (ECL) with West Femto chemiluminescent substrate (Thermo Scientific) using the ChemiDoc MP Imaging System (Bio-Rad Laboratories). Image analysis was performed using Image Lab 5.2 software (Bio-Rad Laboratories).

### 2.11. Flow Cytometry Measurement of Autolysosome Formation

Cells transfected with the Premo™ Autophagy Tandem Sensor RFP-GFP-LC3B Kit were detached from the cell culture plate using TrypLE™ Express (Gibco), washed with ice-cold PBS, and stained with LIVE/DEAD™ Fixable Aqua Dead Cell Stain Kit (Invitrogen) for 30 min on ice following the manufacturer's recommendation. Cells were then washed once with PBS and resuspended in FACS buffer (PBS with 2% FBS and 0.1% NaN_3_). An Attune™ NxT Flow Cytometer (Invitrogen) was used to perform flow cytometry. Data were analyzed using FlowJo software (BD Biosciences). The appropriate forward/side scatter profile was used to selectively include singlet cells in the analysis. Nonviable cells were excluded from the analysis by gating on cells unstained with LIVE/DEAD™ Fixable Aqua Dead Cell Stain. Only the cells expressing the RFP-GFP-LC3B tandem construct (high RFP signal) were included in the analysis.

### 2.12. Immunostaining

In poly-D-lysine-coated 4-well chamber slides (Corning), 50000 cells per chamber were cultured for 48 h, treated, fixed with 4% paraformaldehyde solution for 15 min at 37°C, permeabilized with 0.2% Triton X-100 in PBS for 10 min, and blocked with 3% normal goat serum in PBS for 30 min. Cells were then incubated overnight at 4°C with primary antibodies for LAMP1, LC3B, STX17, or TOMM20 (all 1 : 200) or 3 hours at room temperature for ZO-1 (1 : 100) immunostaining. After washing, cells were incubated with secondary antibodies and Hoechst 33342 (1 *μ*g/mL) for 1 h. Immunostainings were observed using a ZEISS LSM 700 confocal microscope (LAMP1, LC3B, STX17, and TOMM20) or a ZEISS Axio Observer (ZO-1).

### 2.13. Colocalization Analysis

Manders' colocalization coefficient analysis was performed using JACoP (Just Another Colocalization Plugin) in ImageJ software (NIH Image).

### 2.14. Statistical Analysis

Statistical analyses were performed using GraphPad Prism 8.0 software (GraphPad Software). One-way analysis of variance (ANOVA) with Sidak's multiple comparison test was performed after confirming the normality of residuals by the D'Agostino-Pearson test. Data are presented as mean ± standard deviation (SD). A *p* value lower than 0.05 was considered statistically significant. All comparisons made and corresponding *p* values are presented in supplementary materials (Table S2).

## 3. Results

### 3.1. Sodium Iodate Caused a Concentration-Dependent Cytotoxicity on hTERT RPE-1 Cells

**Figure 1:**
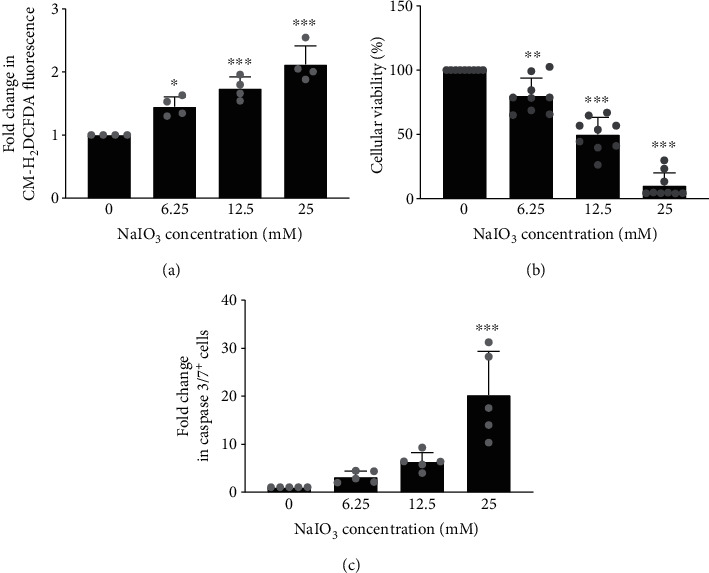
NaIO_3_ induced concentration-dependent ROS formation and death of hTERT RPE-1 cells. hTERT RPE-1 cells were treated with increasing concentrations of NaIO_3_. (a) Cellular ROS after 2 h relative to vehicle-treated cells (*n* = 4). (b) Cellular viability assessed by CCK-8 after 24 h (*n* = 9). (c) Proportion of cells positive for active caspase 3/7 after 4 h presented as fold change relative to vehicle-treated cells (*n* = 5). Mean ± SD, ^∗^*p* < 0.05, ^∗∗^*p* < 0.01, and ^∗∗∗^*p* < 0.001 vs vehicle.

The human telomerase reverse transcriptase subunit-immortalized RPE cell line hTERT RPE-1 has previously been used to study the impact of oxidative stress on RPE cell energy metabolism [30], as well as the cytoprotective role of autophagy in the RPE [31]. Oxidative damage to hTERT RPE-1 cells was induced using NaIO_3_ as oxidant. After treatment with NaIO_3_ for 2 h, the cells exhibited a concentration-dependent increase in ROS accumulation (Figure 1(a)). The colorimetric reaction of CCK-8, a tetrazolium salt used to measure cellular dehydrogenase activities as an indicator of viability [32], revealed a concentration-dependent decrease in cellular viability after 24 h (Figure 1(b)). An increase in the percentage of cells positive for active caspase 3/7 was suggestive of apoptosis and preceded cell death (Figure 1(c)).

### 3.2. MPE-001 Protected hTERT RPE-1 Cells against NaIO_3_-Induced Cytotoxicity

**Figure 2:**
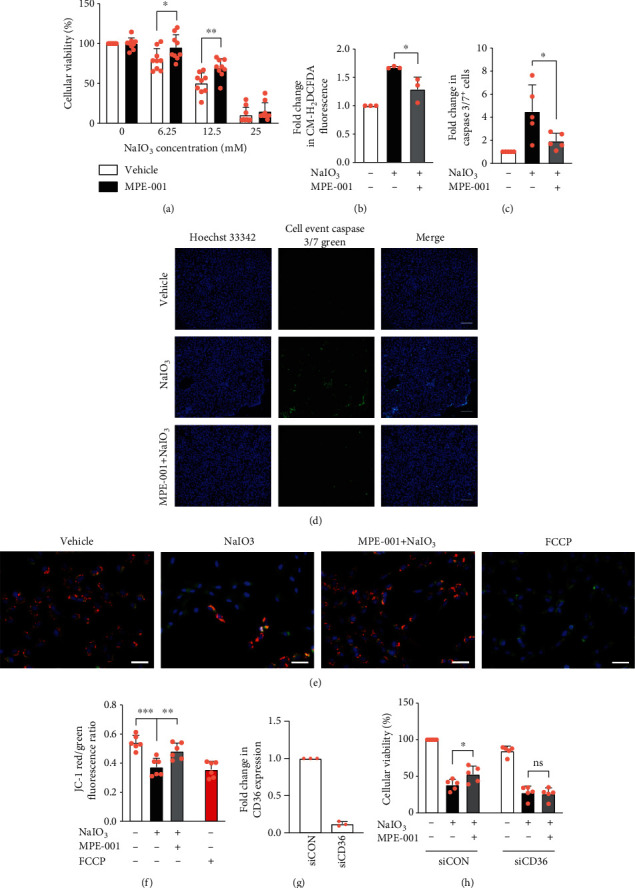
MPE-001 prevented NaIO_3_-induced ROS formation and apoptosis of hTERT RPE-1 cells. hTERT RPE-1 cells were pretreated with 1 *μ*M MPE-001 for 2 h and then exposed to NaIO_3_. (a) Cellular viability assessed by CCK-8 after 24 h of NaIO_3_ treatment (*n* = 9). (b) Cellular ROS after 2 h of 12.5 mM NaIO_3_ treatment presented as fold change relative to vehicle-treated cells (*n* = 3). (c) Proportion of cells positive for active caspase 3/7 after 4 h of 12.5 mM NaIO_3_ treatment presented as fold change relative to vehicle-treated cells (*n* = 5). (d) Representative images of CellEvent Caspase-3/7 Green and Hoechst 33342 staining (scale bar = 200 *μ*m). (e) Representative images of JC-1 staining (JC-1 aggregate: red; JC-1 monomer: green; Hoechst 33342: blue; scale bar = 50 *μ*m). (f) Red to green fluorescence ratio of JC-1 after 4 h of 12.5 mM NaIO_3_ treatment. 5 *μ*M FCCP was used as control (*n* = 6). (g) *CD36* mRNA expression (*n* = 3) and (h) cellular viability assessed by CCK-8 after 24 h of 12.5 mM NaIO_3_ treatment (*n* = 5) of cells incubated for 72 h with either 20 nM control siRNA (siCON) or CD36 siRNA (siCD36). Mean ± SD, ns: nonsignificant, ^∗^*p* < 0.05, ^∗∗^*p* < 0.01, and ^∗∗∗^*p* < 0.001.

The protective effect of the CD36 ligand MPE-001 on RPE cell oxidative damage was ascertained by a pretreatment with the azapeptide for 2 h, followed by exposure to NaIO_3_. Treatment with MPE-001 alone did not impact the colorimetric reaction of CCK-8. MPE-001 improved the viability of hTERT RPE-1 cells exposed to 6.25 and 12.5 mM NaIO_3_ (Figure 2(a)). The 12.5 mM concentration of NaIO_3_, against which MPE-001 had the most significant effect, was employed in subsequent experiments. MPE-001 caused ~0.57-fold and~0.73-fold decreases of NaIO_3_-induced ROS formation (Figure 2(b)) and apoptotic cell death (Figures 2(c) and 2(d)), respectively. The mitochondrial membrane potential is known to be disrupted during early apoptosis [33] and was assessed to confirm the effect of MPE-001 on apoptosis. Monomeric JC-1 dye has a cationic nature and green fluorescence. Upon accumulation in healthy mitochondria, JC-1 forms aggregates which emit a red fluorescence. Treatment with the oxidative phosphorylation uncoupler FCCP as positive control for 4 h caused a decrease in JC-1 red aggregates in all cells, indicative of mitochondrial depolarization. A visible loss of red fluorescence in apoptotic cells was detected after exposure to NaIO_3_ for 4 h. In contrast, red fluorescence persisted and barely any apoptosis was observed in cells pretreated with MPE-001 prior to exposure to the oxidant (Figures 2(e) and 2(f) and Figure S1).

The CD36 dependence of the protective effect of MPE-001 was verified using RNA interference. After 72 h, knockdown with siRNA decreased *CD36* mRNA expression by 88% (Figure 2(g)) and the cytoprotective effect of MPE-001 was completely abolished (Figure 2(h)).

### 3.3. MPE-001 Protected Differentiated ARPE-19 Cells against NaIO_3_-Induced Cytotoxicity

The human cell line ARPE-19 was used to validate the effect of MPE-001 on a differentiated and polarized RPE cell culture model. Culture of confluent ARPE-19 cells in low-serum medium has been shown to recapitulate some characteristics of native RPE cells, including the cobblestone morphology and expression of RPE-specific genes. Moreover, a polarized ARPE-19 cell monolayer can be promoted using the Transwell culture system [29]. Differentiated ARPE-19 cells grown on a Transwell insert displayed lateral distribution of zonal occludens-1 (ZO-1) suggestive of tight junction formation (Figure S2A) and a similar loss of cellular viability to hTERT RPE-1 cells when exposed to 12.5 mM NaIO_3_ for 24 h (Figure S2B). The lower compartment of the Transwell culture system was pretreated with MPE-001, because CD36 expression has been mainly observed at the basal surface of rat RPE cells [34]. MPE-001 protected differentiated ARPE-19 cells from NaIO_3_ and improved cellular viability from 62% to 93% (Figure S2B).

### 3.4. The Protective Effect of MPE-001 Was Not Mediated by a Transcriptional Upregulation of Antioxidant Enzyme Expression

**Figure 3:**
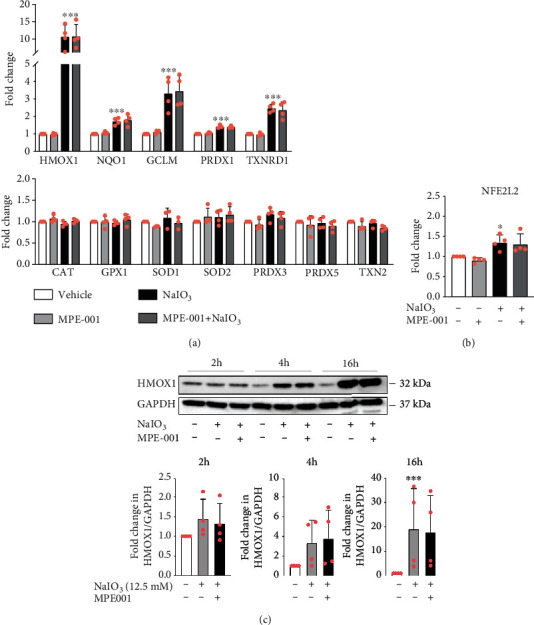
The effect of NaIO_3_ and/or MPE-001 treatment on antioxidant enzyme expression. hTERT RPE-1 cells were pretreated with 1 *μ*M MPE-001 for 2 h and then exposed to 12.5 mM NaIO_3_. (a) mRNA expression of antioxidant enzymes and (b) *NFE2L2* expression after 4 h of NaIO_3_ treatment. Data are presented as fold change relative to vehicle controls. *n* = 3–4, mean ± SD, ^∗^*p* < 0.05 and ^∗∗∗^*p* < 0.001 vs vehicle. (c) Immunoblot of HMOX1 and GAPDH (upper) and relative quantification of HMOX1/GAPDH (lower) at different time points following NaIO_3_ treatment. *n* = 4, mean ± SD, and ^∗∗∗^*p* < 0.001 vs vehicle.

The importance of antioxidant enzyme expression on the antioxidant mechanism of MPE-001 action was ascertained using qRT-PCR. Neither NaIO_3_ nor MPE001 exhibited effect on the expression of *CAT*, *GPX1*, *SOD1*, *SOD2*, *PRDX3*, *PRDX5*, and *TXN2* (Figure 3(a)). Treatment with NaIO_3_ for 4 h increased expression of genes targeted by the transcription factor NRF2 (or NFE2L2): *HMOX1*, *NQO1*, *GCLM*, *PRDX1*, and *TXNRD1* (Figure 3(a)). Moreover, exposure to NaIO_3_ increased expression of *NFE2L2* (Figure 3(b)), which is a master regulator of the antioxidant system [35]. On the contrary, MPE-001 had no effect on the expression of *NFE2L2*, *NFE2L2*-targeted genes, nor HMOX1 protein at different timepoints after NaIO_3_ treatment (Figures 3(a)–3(c)). The protective effect of MPE-001 was therefore not likely mediated by a transcriptional upregulation of antioxidant enzymes in the RPE cell model.

### 3.5. MPE-001 Improved Autophagic Flux in NaIO_3_-Treated hTERT RPE-1 Cells and Its Cytoprotective Effect Was Autophagy Dependent

**Figure 4:**
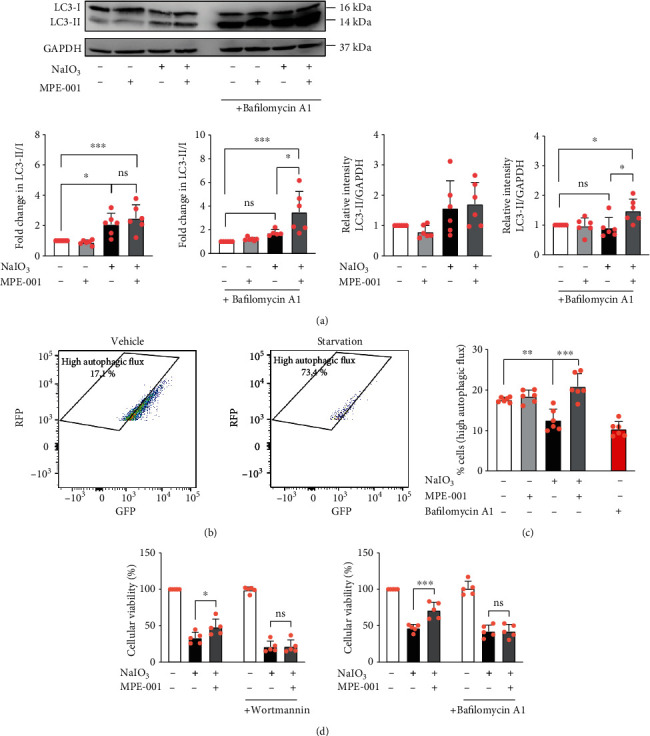
MPE-001 enhanced autophagy in NaIO_3_-treated cells. hTERT RPE-1 cells were pretreated with 1 *μ*M MPE-001 for 2 h and then exposed to 12.5 mM NaIO_3_ for 4 h (a, c, and d) or treated with 10 nM bafilomycin for 6 hours (c) or starved in HBSS for 1 hour (b). (a) Immunoblot of LC3B and GAPDH (upper) and quantification of LC3-II/LC3-I and LC3-II/GAPDH ratio (lower) relative to respective vehicle controls in the absence and presence of 10 nM bafilomycin A1 (*n* = 6). (b, c) Flow cytometry analysis of RFP and GFP signals of cells transfected with an RFP-GFP-LC3B tandem construct. Example of gating used to identify the percentage of cells with high autophagic flux in (b) vehicle-treated and starved cells and its quantification in cells treated with (c) vehicle, MPE-001, and/or NaIO_3_ or with bafilomycin A1 (*n* = 6). (d) Cellular viability assessed by CCK-8 after treatment with NaIO_3_ for 24 h in the absence and presence of 100 nM wortmannin (left, *n* = 5) or 10 nM bafilomycin A1 (right, *n* = 5). Mean ± SD, ns: nonsignificant, ^∗^*p* < 0.05, ^∗∗^*p* < 0.01, and ^∗∗∗^*p* < 0.001.

The relevance of autophagy in the antioxidative and cytoprotective mechanisms of MPE-001 action was next investigated because of the absence of an effect on antioxidant gene expression. Microtubule-associated protein light chain 3B (LC3B) is a structural protein of autophagosomal membranes. During autophagy, the cytosolic form of LC3B (LC3-I) undergoes lipidation to form LC3-II which is integrated into growing phagophores [36]. Consequently, the LC3-II/I ratio is a discerning indicator of autophagy [37]. Treatment with NaIO_3_ for 4 h induced a statistically significant LC3-II/I ratio increase compared to vehicle-treated cells, which was not observed when all cells were concomitantly treated with the inhibitor of lysosomal acidification bafilomycin A1 (Figure 4(a)). The latter LC3-II/I ratios were unchanged and increased by pretreatment with MPE-001 in the absence and presence, respectively, of bafilomycin A1. Relative to vehicle controls, combined treatment with NaIO_3_ and MPE-001 induced 2.49- and 3.50-fold increases in the LC3-II/I ratio in the absence and presence, respectively, of bafilomycin A1 (Figure 4(a)), suggestive of increased autophagosome biogenesis and degradation. Similar trends were observed in the LC3-II/GAPDH ratio (Figure 4(a)). These data indicate an inefficient clearing of autophagosomes in NaIO_3_-treated cells, which is improved by MPE001 pretreatment. MPE-001 treatment alone did not alter the LC3-II/I ratio (Figure 4(a)).

The effects of MPE-001 on autophagic flux were further validated using hTERT RPE-1 cells expressing an RFP-GFP-LC3B tandem construct. To monitor autolysosome formation, the quenching of acid-labile green fluorescent protein (GFP) signal relative to the acid-stable red fluorescent protein (RFP) signal was measured by flow cytometry. Cells with high autophagic flux (high RFP/GFP ratio) were defined using cells starved for 1 hour in Hank's balanced salt solution (HBSS) (Figure 4(b)). Treatment with NaIO_3_ or bafilomycin A1 for 4 hours reduced autophagic flux, decreasing the percentage of cells with high autophagic flux from 18% to 13% or 10%, respectively. Treatment with MPE-001 alone did not induce autolysosome formation but increased the percentage of cells with high autophagic flux in NaIO_3_-exposed cells to 21% (Figure 4(c)).

Autophagy inhibitors wortmannin and bafilomycin A1 both abolished the cytoprotective effect of MPE-001 on hTERT RPE-1 cells (Figure 4(d)). In contrast, bafilomycin A1 had no effect on the improvement of the viability of NaIO_3_-treated cells that was produced by the antioxidant NAC, which is a synthetic precursor of L-cysteine and an elevator of glutathione biosynthesis (Figure S3A, B). The cytoprotective effect of MPE-001 is thus mediated by autophagy and not through the direct scavenging of NaIO_3_-generated ROS. Similarly, the cytoprotective effect of MPE-001 against NaIO_3_ on differentiated ARPE-19 cells was abolished by bafilomycin A1, suggesting an autophagy-dependent protective mechanism in the cultured system (Figure S4).

### 3.6. MPE-001 Improved Autophagosome Maturation in NaIO_3_-Treated hTERT RPE-1 Cells

**Figure 5:**
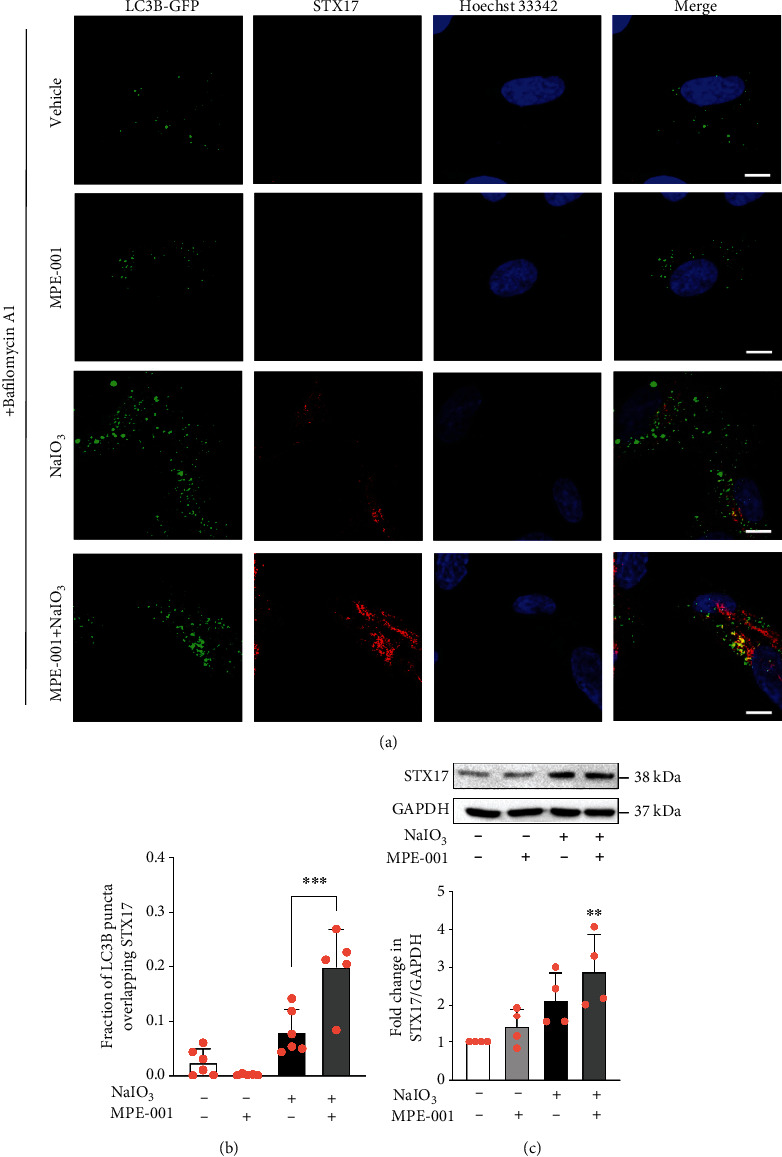
MPE-001 increased the recruitment of STX17 to autophagosomes in NaIO_3_-treated cells. hTERT RPE-1 cells were pretreated with 1 *μ*M MPE-001 for 2 h and then exposed to 12.5 mM NaIO_3_ for 4 h. (a) Representative images of LC3B-GFP-transfected cells immunostained for STX17 following treatments in the presence of 10 nM bafilomycin A1 (scale bar = 10 *μ*m). The same gamma correction was applied to all images. (b) Fraction of LC3B puncta that overlap STX17 staining. Manders' colocalization coefficient analysis was carried out across a series of 5 to 6 images of 6 to 18 cells each. Mean ± SD, ^∗∗∗^*p* < 0.001. (c) Immunoblot of STX17 and GAPDH (upper) and relative quantification of STX17/GAPDH (lower). *n* = 4, mean ± SD, ns: nonsignificant, and ^∗∗^*p* < 0.01 vs vehicle.

Syntaxin 17 (STX17) is a soluble *N*-ethylmaleimide-sensitive factor attachment protein receptor (SNARE) required for autophagosome-lysosome fusion. Mature autophagosomes that are primed to fuse with lysosomes are LC3B and syntaxin 17 (STX17) positive [38]. The restorative effect of MPE-001 on NaIO_3_-induced inhibition of autophagic flux was examined in transfected cells expressing LC3B-GFP by immunostaining for STX17. Colocalization of LC3B-GFP puncta and STX17 immunostaining assessed in the presence of bafilomycin A1 was scarce in NaIO_3_-treated cells (Figure 5(a)), indicating a defect in autophagosomal maturation. The proportion of LC3B puncta that colocalize with STX-17 immunostaining was significantly increased in MPE-001-pretreated cells (Figures 5(a) and 5(b)). The NaIO_3_ treatment resulted in STX17 accumulation outside of autophagosomes (Figure 5(a)). Western blotting indicated that the increase in STX17 expression was only statistically significant in the MPE-001-pretreated cells exposed to NaIO_3_ (Figure 5(c)).

### 3.7. MPE-001 Prevented NaIO_3_-Induced Mitochondrial Superoxide Production through Autophagy Enhancement

**Figure 6:**
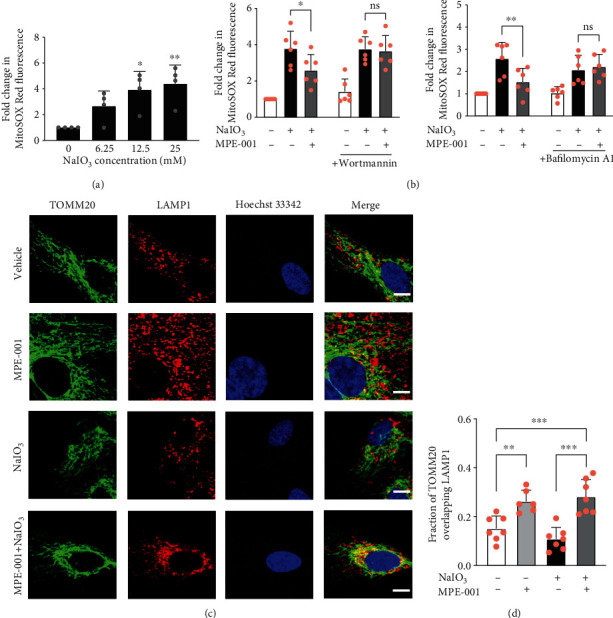
MPE-001 prevented NaIO_3_-induced mitochondrial superoxide formation in an autophagy-dependent manner. (a) Mitochondrial superoxide formation in hTERT RPE-1 cells treated with increasing concentrations of NaIO_3_ for 2 h as fold change relative to vehicle-treated cells (*n* = 4). ^∗^*p* < 0.05 and ^∗∗^*p* < 0.01 vs vehicle. (b, c) Cells were pretreated with 1 *μ*M MPE-001 for 2 h and then exposed to 12.5 mM NaIO_3_. (b) Mitochondrial superoxide formation in the absence and presence of 100 nM wortmannin (left, *n* = 6) or 10 nM bafilomycin A1 (right, *n* = 6) after 2 h of NaIO_3_ treatment. Mean ± SD, ns: nonsignificant, ^∗^*p* < 0.05, and ^∗∗^*p* < 0.01. (c) Representative images of TOMM20 and LAMP1 immunostaining after 4 h of NaIO_3_ treatment (scale bar = 10 *μ*m). (d) Fraction of TOMM20 staining that overlaps with LAMP1 staining. Manders' colocalization coefficient analysis was carried out across a series of 6 to 7 images of 1 to 13 cells each. Mean ± SD, ^∗∗^*p* < 0.01, and ^∗∗∗^*p* < 0.001.

Mitochondria are both a target and a major source of ROS. Improper removal of damaged mitochondria by autophagy results in excessive generation of ROS [39], which may be the causative agent in NaIO_3_-induced cell death. The effects of NaIO_3_ and MPE-001 on mitochondrial ROS formation were examined by measuring superoxide formation. After treatment with NaIO_3_ for 2 h, mitochondrial superoxide increased in a concentration-dependent manner (Figure 6(a)). Pretreatment with MPE-001 decreased the formation of mitochondrial superoxide induced by 12.5 mM NaIO_3_ (Figure 6(b)). Wortmannin and bafilomycin A1 both abolished the effect of MPE-001 on mitochondrial superoxide formation induced by NaIO_3_ (Figure 6(b)), indicating that the activity on ROS was secondary to the influence on autophagy. Immunostaining of the lysosomal-associated membrane protein 1 (LAMP1) and the translocase of outer mitochondrial membrane 20 (TOMM20) showed that MPE-001 treatment increased the colocalization of TOMM20-positive structures with LAMP1-positive structures, regardless of cell exposure to NaIO_3_ (Figures 6(c) and 6(d)). Immunostaining of LC3B and TOMM20 in the presence of bafilomycin A1 showed in contrast that NaIO_3_ treatment, combined or not with MPE-001, increased the recruitment of autophagosomes to mitochondria (Figure S5A, B), indicating that the oxidant inhibits autophagy after earlier steps of phagophore formation, elongation, and cargo engulfment.

The role of decreased mitochondrial ROS for the enhancement of survival of NaIO_3_-treated cells was explored using two mitochondria-selective ROS scavengers: MitoQ and MitoTEMPO, which are thought to insert into the inner mitochondrial membrane [40] and to cross the membrane and accumulate in the mitochondrial matrix, respectively [41]. Pretreatment with MitoTEMPO for 1 h failed to decrease mitochondrial superoxide formation (Figure S6A) and to prevent cell loss (Figure S6B). Pretreatment with MitoQ for 1 h decreased NaIO_3_-induced mitochondrial superoxide formation by ~0.32-fold (Figure S6C) but failed to improve cellular viability (Figure S6D). These results suggest that scavenging of mitochondrial ROS is insufficient to rescue RPE cells from NaIO_3_-induced cytotoxicity.

## 4. Discussion

A regiment composed of antioxidant supplements (vitamins C and E, lutein, and zeaxanthin), zinc, and copper is currently recommended to AMD patients in an attempt to limit oxidative damage to the RPE and preserve photoreceptor integrity [42]. Such a regiment has however limited effect, except in advanced stages [43, 44], and cannot prevent disease onset [45]. New approaches are urgently needed for preventing and halting the pathology of AMD ideally at the earlier stages. CD36 is a promising therapeutic target due in part to the involvement in sterile inflammation and lipid deposition observed in dry AMD [46]. Previously, GHRP-6 derivatives have demonstrated beneficial effects as CD36 ligands that preserve photoreceptors in mouse models of AMD [18, 20]. The potential of such CD36 ligands to mitigate oxidative stress and preserve RPE integrity has now been demonstrated.

The cytoprotective effect of the GHRP-6 derivative CD36 ligand MPE-001 against NaIO_3_-induced oxidative damage was revealed in human RPE cells in culture. The oxidant was shown to disrupt autophagic flux by a mechanism that entailed autophagosome formation without lysosome fusion. In spite the activation of the antioxidant system, oxidative stress and cell death persisted likely due to the inability of RPE cells to clear damaged proteins and organelles. The NaIO_3_-induced defect in autophagic flux was remedied by pretreatment of cells with MPE-001. Improvement in autophagic processes by MPE-001 resulted in decreased mitochondrial ROS accumulation and diminished apoptosis of RPE cells. In unstressed cells, MPE-001 treatment had no effect on the antioxidant system nor on autophagy.

Apoptosis mediated by caspase 3/7/8 activation [23, 27] and necroptosis [22, 27, 28] both have been reported to account for RPE cell death in culture treated with NaIO_3_. In accordance with the earlier studies, NaIO_3_ induced caspase 3/7 activation in hTERT RPE-1 cells. Moreover, NaIO_3_-induced cell death was accompanied by a disruption of the mitochondrial membrane potential as shown previously using JC-1 ratio-metric analysis and imaging [23, 26]. MPE-001 was found to decrease NaIO_3_-induced ROS formation and specifically block mitochondrial depolarization and caspase 3/7-mediated apoptosis.

Strategies to boost the antioxidant system in RPE cells have been proposed to treat AMD [26, 47, 48]. For example, activation of NRF2 has been shown to protect RPE cells from oxidative stress both *in vitro* [26] and *in vivo* [49]. In contrast, MPE-001 did not upregulate antioxidant enzyme expression at the studied time points. The effect of MPE-001 on ROS formation was observed as early as 2 h after NaIO_3_ treatment, opposing a mechanism implicating *de novo* transcription of antioxidant enzymes.

Autophagy has been shown to mitigate the impact of oxidative stress in the RPE. After NaIO_3_ treatment, markers of autophagy increased in ARPE-19 cells [24–27] and enhanced autophagic flux protected RPE cells from oxidative stress induced by NaIO_3_ [26] and H_2_O_2_ [50]. Blockade of autophagy by 3-methyladenine or respective knockdowns of *ATG7* and *Beclin-1* all have been observed to exacerbate the oxidative and cytotoxic effects of H_2_O_2_ [50]. In sum, autophagy has been shown to play a crucial role in RPE cell defense against oxidative damage.

MPE-001 protected RPE cells against NaIO_3_-induced oxidative damage by improving autophagic processes. The effects of MPE-001 were completely abolished by wortmannin or bafilomycin A1. Only after complete autophagosome closure does the autophagosome outer membrane recruit STX17, which is not observed in intermediate unclosed states [51]. Moreover, depletion of *STX17* caused an accumulation of autophagosomes without degradation [51]. In this study, few autophagosomes were STX17 positive in NaIO_3_-treated cells, indicating a defect in autophagosome maturation and fusion to lysosomes. Pretreatment with MPE-001 remedied the effect of NaIO_3_ on autophagosome maturation and increased the proportion of LC3B puncta that colocalize with STX17 immunostaining. A role of CD36 in autophagy promotion by secreted glycoprotein CD5 antigen-like protein (CD5L) has been reported in macrophages [52] and in a model of hepatic ischemic/reperfusion injury [53]. In the latter, CD5L was shown to have antiapoptotic and antioxidative effects in a CD36- and Atg7-dependent manner [53]. The CD36 ligand CD5L is expressed in RPE cells and a high amount of circulating CD5L has been observed in some AMD patients [54]. The roles of CD5L in normal RPE cell physiology and AMD pathology have yet to be elucidated.

Increased mitochondrial superoxide formation has previously been associated with the cytotoxic effect of NaIO_3_ [23] as observed in this study. MPE-001 decreased NaIO_3_-induced mitochondrial superoxide production in an autophagy-dependent manner. On the contrary, scavenging of mitochondrial ROS by MitoQ did not rescue hTERT RPE-1 cells. Restored autophagic flux with clearance of defective mitochondria and cytosolic damaged proteins may account for the preventive effect of MPE-001 on mitochondrial depolarization and apoptosis which was likely not attained by the ROS scavenger MitoQ. Autophagy inhibition by bafilomycin A1 in combination with *tert*-butyl hydroperoxide-induced mitochondrial oxidative insult, but not bafilomycin A1 alone, has previously been shown to induce mitochondrial depolarization and cell death [55]. Autophagic processes are therefore crucial in maintaining mitochondrial health in cells subjected to oxidative stress. Curiously, MPE-001 treatment alone increased the colocalization of TOMM20-positive mitochondria with LAMP1-positive lysosomes without increasing TOMM20 colocalization with LC3B. Treatment of the cells with MPE-001 might be accelerating mitochondria turnover in healthy cells in an autophagy-independent anner. Impaired lysosomal removal of damaged mitochondria has been theorized to contribute to cell death associated with aging [56]. Moreover, mitochondria in the RPE of AMD patients exhibit more pronounced age-associated alterations in morphology and activity [57, 58]. The improved autophagic activity resulting from CD36 activation may have broad benefits in a wide range of age-related diseases including AMD.

## 5. Conclusion

**Figure 7:**
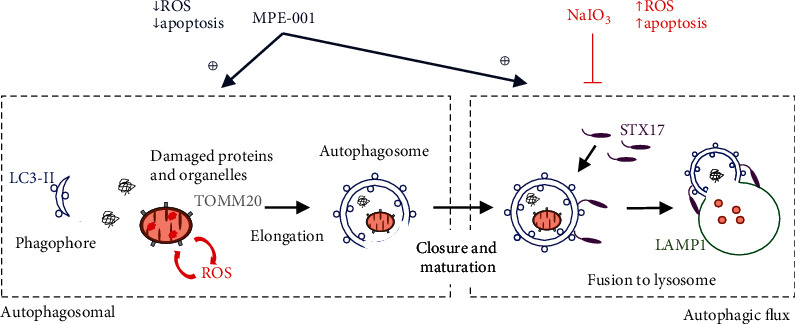
Underlying mechanism of the antioxidative and antiapoptotic effects of CD36 activation by MPE-001. Treatment of RPE cells with NaIO_3_ disrupts later steps of autophagic processes, leading to cell death. MPE-001 decreases ROS formation and apoptosis by promoting the closure and maturation of autophagosomes, which will recruit STX17 and fuse with lysosomes for the degradation of damaged proteins and organelles.

In summary, the CD36 ligand MPE-001 had antioxidative and antiapoptotic effects on RPE cells by a mechanism featuring restoration of autophagic flux (Figure 7). Modulation of the RPE redox status by pharmacological targeting of CD36 was evidenced for the first time. Combined with their known anti-inflammatory effect, the ability of azapeptide CD36 ligands to modulate oxidative stress offers potential for preserving RPE and photoreceptor integrity and a novel strategy for remedying the pathogenesis of dry AMD.

## Data Availability

The data used to support the findings of this study are available upon request.

## Supplementary Materials

Supplementary materials contains Tables S1-S2 and Figures S1-S6. Table S1: primer sequences. Table S2: statistical comparisons and *p* values. Figure S1: MPE-001 pretreatment prevents the NaIO_3_-induced loss of mitochondrial membrane potential. Figure S2: MPE-001 improved the viability of differentiated ARPE-19 cells exposed to NaIO_3_. Figure S3: NAC protected hTERT RPE-1 cells against NaIO_3_-induced cytotoxicity in an autophagy-independent manner. Figure S4: bafilomycin A1 abolished the cytoprotective effect of MPE-001 on differentiated ARPE-19 cells. Figure S5: the effect of MPE-001 and/or NaIO_3_ treatments on LC3B and TOMM20 colocalization. Figure S6: the effect of mitochondria-selective antioxidant compounds on NaIO_3_-induced cytotoxicity.
