# Supplementary material for: The CD36 Ligand-Promoted Autophagy Protects Retinal Pigment Epithelial Cells from Oxidative Stress
Source: Oxid Med Cell Longev. 2021 Mar 28;2021:6691402. doi: 10.1155/2021/6691402 (PMC8019622; doi:10.1155/2021/6691402)
Supplement: Supplementary materials — contains Tables S1-S2 and Figures S1-S6. Table S1: primer sequences. Table S2: statistical comparisons and p values. Figure S1: MPE-001 pretreatment prevents the NaIO3-induced loss of mitochondrial membrane potential. Figure S2: MPE-001 improved the viability of differentiated ARPE-19 cells exposed to NaIO3. Figure S3: NAC protected hTERT RPE-1 cells against NaIO3-induced cytotoxicity in an autophagy-independent manner. Figure S4: bafilomycin A1 abolished the cytoprotective effect of MPE-001 on differentiated ARPE-19 cells. Figure S5: the effect of MPE-001 and/or NaIO3 treatments on LC3B and TOMM20 colocalization. Figure S6: the effect of mitochondria-selective antioxidant compounds on NaIO3-induced cytotoxicity. [file 6691402.f1.pdf]

Table S1. Primer sequences

Gene symbol	Gene name	Forward	Reverse
CAT	Catalase	gctcattttgaccgagagaga	tgacctcaaagtagccaaagg
CD36	Cluster of differentiation 36	tggaacagaggctgacaactt	ttgattttgatagatatgggatgc
GAPDH	Glyceraldehyde-3-phosphate dehydrogenase	gagccaaaagggtcatcatctc	aggaggcattgctgatgatct
GCLM	Glutamate-cysteine ligase modifier subunit	gacaaaacacagttggaacagc	cagtcaaatctggtggcatc
GPX1	Glutathione peroxidase 1	cgccaagaacgaagagattc	gttcacctcgcacttctcg
HMOX1	Heme oxygenase 1	ggcagagggtgatagaagagg	agctcctgcaactcctcaaa
NFE2L2	Nuclear factor, erythroid 2 like 2	acacggtccacagctcatc	tgcctccaaagtatgtcaatca
NQO1	NAD(P)H quinone dehydrogenase 1	cagctcaccgagagcctagt	gagtgagccagtacgatcagtg
PPIA	Peptidylprolyl isomerase A	cctaaagcatacgggtcctg	tttcactttgccaaacacca
PRDX1	Peroxiredoxin 1	aggccttccagttcactgac	tttgctcttttggacatcagg
PRDX3	Peroxiredoxin 3	agcaaaattattcagcaccagtt	ccttaaaatagggtgcatgctg
PRDX5	Peroxiredoxin 5	tcctggctgatcccactg	atgccatcctgtaccaccat
SOD1	Superoxide dismutase 1	tccatgttcatgagtttggagat	cccaccgtgttttctggata
SOD2	Superoxide dismutase 2	aatcaggatccactgcaagg	taagcgtgctcccacacat
TXN2	Thioredoxin 2	gagacaccagtggttgtgga	ttggccaccatcttctctaac
TXNRD1	Thioredoxin reductase 1	caattggaatccaccctgtc	ccacactggggcttaacct
YWHAZ	Tyrosine 3-monooxygenase/tryptophan 5-monooxygenase activation protein zeta	gcaattactgagagacaacttgaca	tggaaggccggttaatttt

Table S2. Statistical comparisons and p-values

	Pair compared		Adjusted p-value
**Figure 1A**			
	$NaIO_3$ 0 mM	$NaIO_3$ 6.25 mM	0.0161
	$NaIO_3$ 0 mM	$NaIO_3$ 12.5 mM	0.0004
	$NaIO_3$ 0 mM	$NaIO_3$ 25 mM	<0.0001
**Figure 1B**			
	$NaIO_3$ 0 mM	$NaIO_3$ 6.25 mM	<0.0012
	$NaIO_3$ 0 mM	$NaIO_3$ 12.5 mM	<0.0001
	$NaIO_3$ 0 mM	$NaIO_3$ 25 mM	<0.0001
**Figure 1C**			
	$NaIO_3$ 0 mM	$NaIO_3$ 6.25 mM	0.8518
	$NaIO_3$ 0 mM	$NaIO_3$ 12.5 mM	0.2394
	$NaIO_3$ 0 mM	$NaIO_3$ 25 mM	<0.0001
**Figure 2A**			
	$NaIO_3$ 0 mM	$NaIO_3$ 6.25 mM	0.0029
	$NaIO_3$ 0 mM	$NaIO_3$ 12.5 mM	<0.0001
	$NaIO_3$ 0 mM	$NaIO_3$ 25 mM	<0.0001
	$NaIO_3$ 0 mM	MPE-001 + $NaIO_3$ 0 mM	>0.9999
	$NaIO_3$ 6.25 mM	MPE-001 + $NaIO_3$ 6.25 mM	0.0477
	$NaIO_3$ 12.5 mM	MPE-001 + $NaIO_3$ 12.5 mM	0.0042
	$NaIO_3$ 25 mM	MPE-001 + $NaIO_3$ 25 mM	0.9723
**Figure 2B**			
	Vehicle	$NaIO_3$	0.0011
	$NaIO_3$	MPE-001 + $NaIO_3$	0.0188
**Figure 2C**			
	Vehicle	$NaIO_3$	0.0037
	$NaIO_3$	MPE-001 + $NaIO_3$	0.0261
**Figure 2F**			
	Vehicle	$NaIO_3$	0.0002
	$NaIO_3$	MPE-001 + $NaIO_3$	0.0075
**Figure 2H**			
siCON	Vehicle	$NaIO_3$	<0.0001
	$NaIO_3$	MPE-001 + $NaIO_3$	0.0304
siCD36	Vehicle	$NaIO_3$	<0.0001
	$NaIO_3$	MPE-001 + $NaIO_3$	0.9951
**Figure 3A**			
HMOX1	Vehicle	MPE-001	>0.9999
	Vehicle	$NaIO_3$	0.0003

	$NaIO_3$	MPE-001+ $NaIO_3$	0.9999
**Figure 3A**			
NQO1	Vehicle	MPE-001	0.9596
	Vehicle	$NaIO_3$	0.0005
	$NaIO_3$	MPE-001+ $NaIO_3$	0.9266
**Figure 3A**			
GCLM	Vehicle	MPE-001	0.9442
	Vehicle	$NaIO_3$	0.0009
	$NaIO_3$	MPE-001+ $NaIO_3$	0.9442
**Figure 3A**			
PRDX 1	Vehicle	MPE-001	0.8235
	Vehicle	$NaIO_3$	0.0002
	$NaIO_3$	MPE-001+ $NaIO_3$	0.9607
**Figure 3A**			
TXNR D1	Vehicle	MPE-001	0.9987
	Vehicle	$NaIO_3$	<0.0001
	$NaIO_3$	MPE-001+ $NaIO_3$	0.9293
**Figure 3A**			
CAT	Vehicle	MPE-001	0.2255
	Vehicle	$NaIO_3$	0.2255
	$NaIO_3$	MPE-001+ $NaIO_3$	0.2255
**Figure 3A**			
GPX1	Vehicle	MPE-001	0.9100
	Vehicle	$NaIO_3$	0.9100
	$NaIO_3$	MPE-001+ $NaIO_3$	0.6008
**Figure 3A**			
SOD1	Vehicle	MPE-001	0.6876
	Vehicle	$NaIO_3$	0.8048
	$NaIO_3$	MPE-001+ $NaIO_3$	0.6601
**Figure 3A**			
SOD2	Vehicle	MPE-001	0.6639
	Vehicle	$NaIO_3$	0.7285
	$NaIO_3$	MPE-001+ $NaIO_3$	0.9463
**Figure 3A**			
PRDX 3	Vehicle	MPE-001	0.5645
	Vehicle	$NaIO_3$	0.1908
	$NaIO_3$	MPE-001+ $NaIO_3$	0.5645
**Figure 3A**			
PRDX 5	Vehicle	MPE-001	0.8548
	Vehicle	$NaIO_3$	0.8548
	$NaIO_3$	MPE-001+ $NaIO_3$	0.8548

**Figure 3A**			
TXN2	Vehicle	MPE-001	0.1318
	Vehicle	$NaIO_3$	0.5323
	$NaIO_3$	MPE-001+ $NaIO_3$	0.1148
**Figure 3B**			
	Vehicle	MPE-001	0.8258
	Vehicle	$NaIO_3$	0.0301
	$NaIO_3$	MPE-001+ $NaIO_3$	0.9812
**Figure 3C**			
2h	Vehicle	$NaIO_3$	>0.9999
	$NaIO_3$	MPE-001+ $NaIO_3$	>0.9999
**Figure 3C**			
4h	Vehicle	$NaIO_3$	0.9985
	$NaIO_3$	MPE-001+ $NaIO_3$	>0.9999
**Figure 3C**			
16h	Vehicle	$NaIO_3$	0.0125
	$NaIO_3$	MPE-001+ $NaIO_3$	>0.9999
**Figure 4A LC3-II/I**			
	Vehicle	MPE-001	0.9962
	Vehicle	$NaIO_3$	0.0159
	Vehicle	MPE-001+ $NaIO_3$	0.0010
	$NaIO_3$	MPE-001+ $NaIO_3$	0.6748
**Figure 4A LC3-II/I**			
+Bafilomycin A1	Vehicle	MPE-001	0.9907
	Vehicle	$NaIO_3$	0.5643
	Vehicle	MPE-001+ $NaIO_3$	0.0005
	$NaIO_3$	MPE-001+ $NaIO_3$	0.0189
**Figure 4A LC3-II/GAPDH**			
	Vehicle	MPE-001	0.96
	Vehicle	$NaIO_3$	0.3555
	Vehicle	MPE-001+ $NaIO_3$	0.1759
	$NaIO_3$	MPE-001+ $NaIO_3$	0.9902
**Figure 4A LC3-II/GAPDH**			
+Bafilomycin A1	Vehicle	MPE-001	0.9996
	Vehicle	$NaIO_3$	0.9727
	Vehicle	MPE-001+ $NaIO_3$	0.0123
	$NaIO_3$	MPE-001+ $NaIO_3$	0.0414
**Figure 4C**			
	Vehicle	MPE-001	0.9726
	Vehicle	$NaIO_3$	0.0024
	Vehicle	MPE-001+ $NaIO_3$	0.0722

$NaIO_3$	MPE-001+ $NaIO_3$	<0.0001
**Figure 4D Left**		
Vehicle	Wortmannin	>0.9999
Vehicle	$NaIO_3$	<0.0001
$NaIO_3$	MPE-001 + $NaIO_3$	0.0259
Wortmannin	$NaIO_3$ + Wortmannin	<0.0001
$NaIO_3$ + Wortmannin	MPE-001 + $NaIO_3$ + Wortmannin	>0.9999
**Figure 4D Right**		
Vehicle	Bafilomycin A1	0.9997
Vehicle	$NaIO_3$	<0.0001
$NaIO_3$	MPE-001 + $NaIO_3$	0.0004
Bafilomycin A1	$NaIO_3$ + Bafilomycin A1	<0.0001
$NaIO_3$ + Bafilomycin A1	MPE-001 + $NaIO_3$ + Bafilomycin A1	>0.9999
**Figure 5B**		
Vehicle	MPE-001	0.8481
Vehicle	$NaIO_3$	0.1137
Vehicle	MPE-001+ $NaIO_3$	<0.0001
$NaIO_3$	MPE-001+ $NaIO_3$	0.0006
**Figure 5C**		
Vehicle	MPE-001	0.7872
Vehicle	$NaIO_3$	0.0923
Vehicle	MPE-001+ $NaIO_3$	0.0045
**Figure 6A**		
$NaIO_3$ 0 mM	$NaIO_3$ 6.25 mM	0.1925
$NaIO_3$ 0 mM	$NaIO_3$ 12.5 mM	0.0126
$NaIO_3$ 0 mM	$NaIO_3$ 25 mM	0.0045
**Figure 6B Left**		
Vehicle	Wortmannin	0.8714
Vehicle	$NaIO_3$	<0.0001
$NaIO_3$	MPE-001 + $NaIO_3$	0.0458
Wortmannin	$NaIO_3$ + Wortmannin	<0.0001
$NaIO_3$ + Wortmannin	MPE-001 + $NaIO_3$ + Wortmannin	0.9996
**Figure 6B Right**		
Vehicle	Bafilomycin A1	>0.9999
Vehicle	$NaIO_3$	<0.0001
$NaIO_3$	MPE-001 + $NaIO_3$	0.0095
Bafilomycin A1	$NaIO_3$ + Bafilomycin A1	0.0105
$NaIO_3$ + Bafilomycin A1	MPE-001 + $NaIO_3$ + Bafilomycin A1	0.9931

**Figure 6D**			
	Vehicle	MPE-001	0.0050
	Vehicle	$NaIO_3$	0.4974
	Vehicle	MPE-001+ $NaIO_3$	0.0007
	$NaIO_3$	MPE-001+ $NaIO_3$	<0.0001
**Figure S2B**			
	Vehicle	MPE-001	0.9999
	Vehicle	$NaIO_3$	0.0010
	$NaIO_3$	MPE-001+ $NaIO_3$	0.0361
**Figure S3A**			
	Vehicle	NAC	0.9794
	Vehicle	$NaIO_3$	<0.0001
	$NaIO_3$	NAC+ $NaIO_3$	0.0374
**Figure S3B**			
	Vehicle	Bafilomycin A1	0.9207
	Vehicle	$NaIO_3$	<0.0001
	$NaIO_3$	NAC + $NaIO_3$	0.0369
	Bafilomycin A1	$NaIO_3$ + Bafilomycin A1	<0.0001
	$NaIO_3$ + Bafilomycin A1	NAC + $NaIO_3$ + Bafilomycin A1	0.0387
**Figure S4**			
	Vehicle	Bafilomycin A1	0.9989
	Vehicle	$NaIO_3$	<0.0001
	$NaIO_3$	MPE-001 + $NaIO_3$	0.0423
	Bafilomycin A1	$NaIO_3$ + Bafilomycin A1	<0.0001
	$NaIO_3$ + Bafilomycin A1	MPE-001 + $NaIO_3$ + Bafilomycin A1	0.9999
**Figure S5B**			
	Vehicle	MPE-001	0.4804
	Vehicle	$NaIO_3$	0.0013
	Vehicle	MPE-001+ $NaIO_3$	0.0112
	$NaIO_3$	MPE-001+ $NaIO_3$	0.7070
**Figure S6A**			
	Vehicle	$NaIO_3$	0.0044
	$NaIO_3$	MitoTEMPO+ $NaIO_3$	0.8997
**Figure S6B**			
	Vehicle	MitoTEMPO	0.0926
	Vehicle	$NaIO_3$	<0.0001
	$NaIO_3$	MitoTEMPO+ $NaIO_3$	0.6774
**Figure S6C**			
	Vehicle	$NaIO_3$	<0.0001
	$NaIO_3$	MitoQ+ $NaIO_3$	0.0364

**Figure S6D**		
Vehicle	MitoQ	0.6014
Vehicle	$NaIO_3$	<0.0001
$NaIO_3$	MitoQ+ $NaIO_3$	0.7568

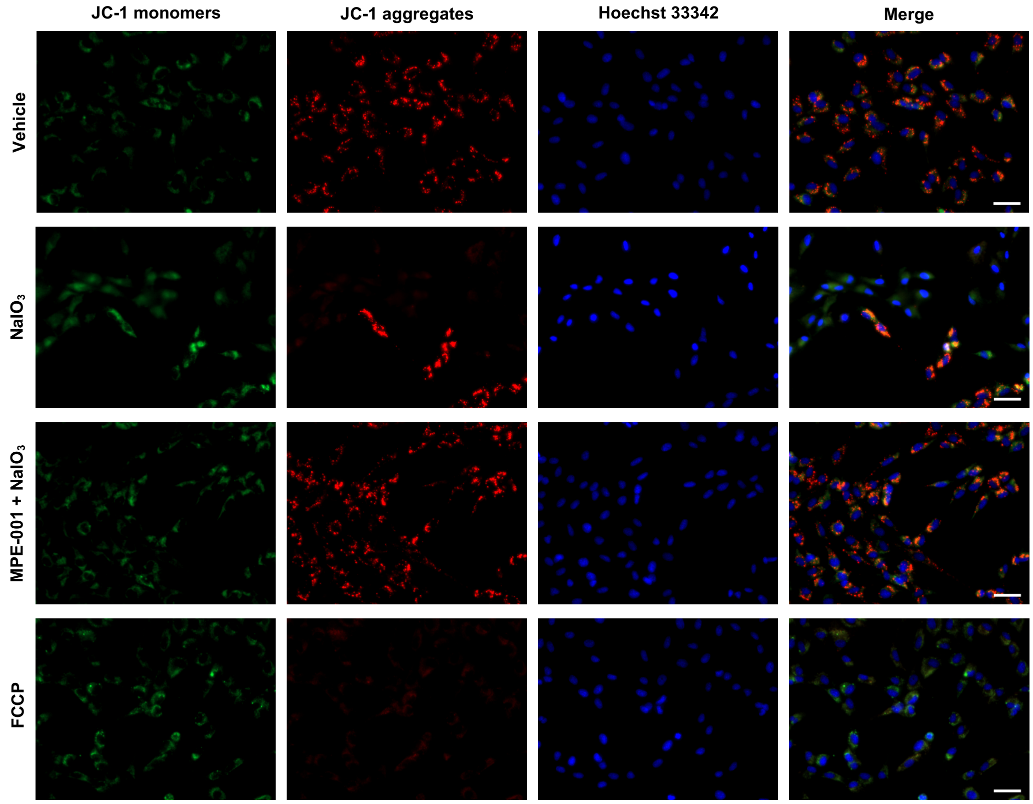

Figure S1. MPE-001 pretreatment prevents the $NaIO_3$-induced loss of mitochondrial membrane potential. hTERT RPE-1 cells were pretreated with 1 μM MPE-001 for 2 h and then exposed to 12.5 mM $NaIO_3$ for 4 h or were treated with 5 μM FCCP for 4 h. Representative images of JC-1 staining are shown (scale bar = 50 μm).

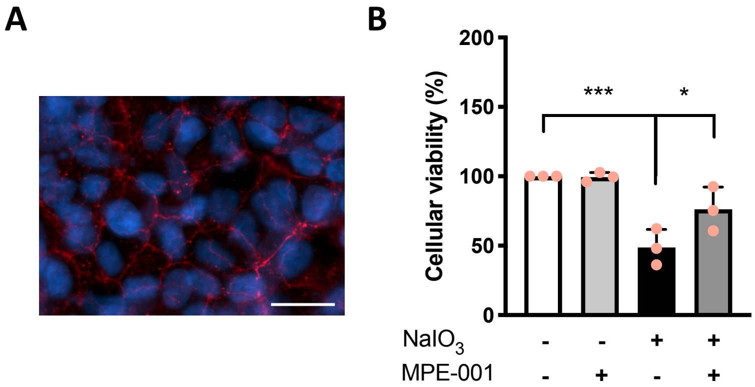

Figure S2. MPE-001 improved the viability of differentiated ARPE-19 cells exposed to $NaIO_3$. (A) Representative image of ZO-1 (red) and Hoechst 33342 (blue) staining of differentiated ARPE-19 cells grown on Transwell inserts (scale bar = 20 µm). (B) Differentiated ARPE-19 cells on Transwell inserts were pretreated with 10 nM MPE-001 through the lower compartment for 2 hours and then exposed to 12.5 mM $NaIO_3$ for 24 hours. Cellular viability was assessed by CCK-8 (n=3). Mean ± SD, * $p < 0.05$, *** $p < 0.001$.

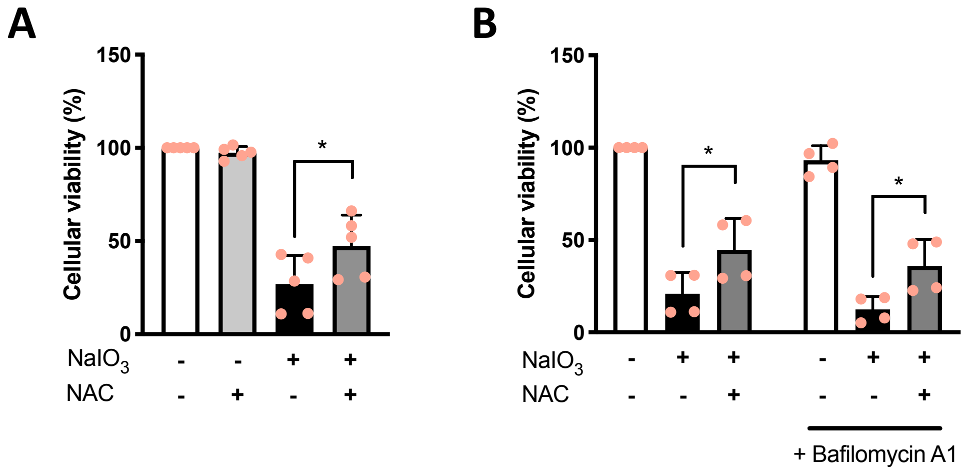

Figure S3. NAC protected hTERT RPE-1 cells against $NaIO_3$-induced cytotoxicity in an autophagy-independent manner. hTERT RPE-1 cells were co-treated with 1.25 mM NAC and $NaIO_3$ for 24 hours. (A) Cellular viability assessed by CCK-8 in the absence (n=5) and (B) in the absence/presence of 10 nM bafilomycin A1 (n=4). Mean ± SD, ns = non-significant, * $p < 0.05$.

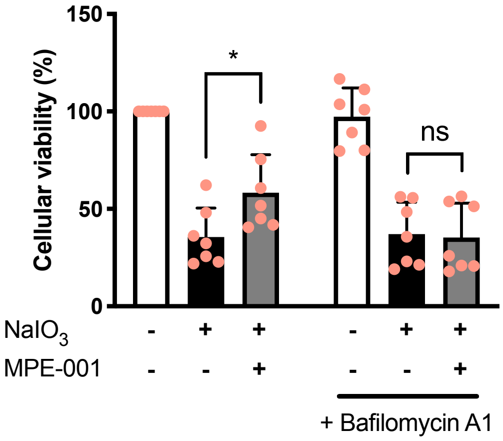

Figure S4. Bafilomycin A1 abolished the cytoprotective effect of MPE-001 on differentiated ARPE-19 cells. Differentiated ARPE-19 cells on Transwell inserts were pretreated with 10 nM MPE-001 through the lower compartment for 2 hours and then exposed to 12.5 mM $NaIO_3$ for 24 hours in the presence/absence of 10 nM bafilomycin A1. Cellular viability was assessed by CCK-8 (n=7), Mean ± SD, ns = non-significant, * $p < 0.05$.

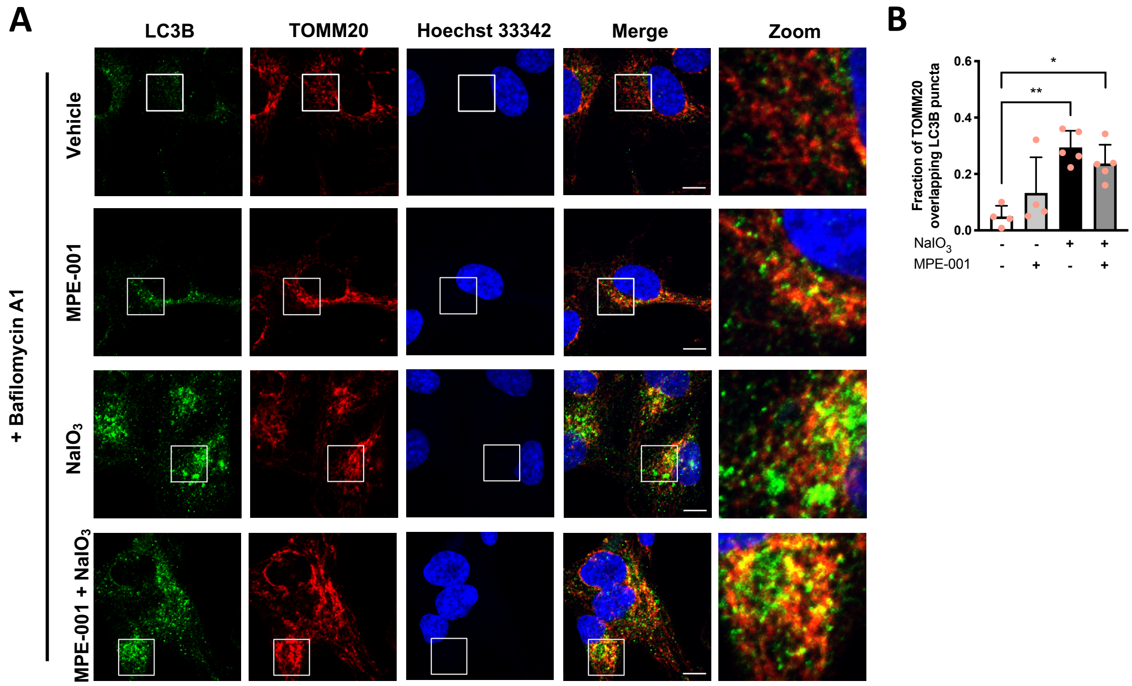

Figure S5. The effect of MPE-001 and/or $NaIO_3$ treatments on LC3B and TOMM20 co-localization. hTERT RPE-1 cells were pretreated with 1 µM MPE-001 for 2 h and then exposed to 12.5 mM $NaIO_3$ for 4 h in the presence of 10 nM bafilomycin A1. (A) Representative images of LC3B and TOMM20 immunostaining are shown (scale bar = 10 µm). The same gamma correction was applied to all images. (B) Manders' colocalization coefficient analysis was carried out across series of 4 to 5 images of 4 to 18 cells each. Mean ± SD, * $p < 0.05$. ** $p < 0.01$.

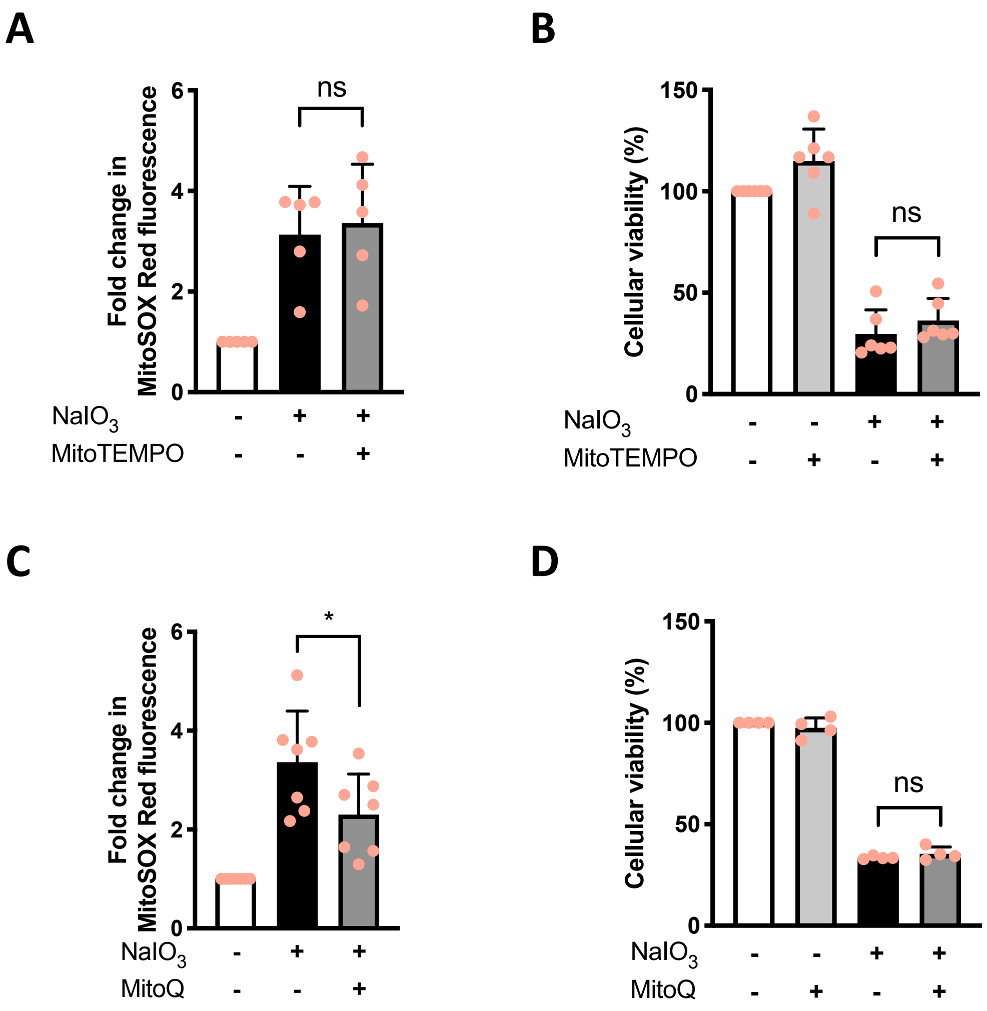

Figure S6. The effect of mitochondria-selective antioxidant compounds on $NaIO_3$-induced cytotoxicity. (A and B) Cells were pretreated with 20 µM MitoTEMPO for 1 hour and then exposed to 12.5 mM $NaIO_3$. (A) Mitochondrial superoxide formation after 2 hours of $NaIO_3$ treatment relative to vehicle-treated cells (n=5) and (B) cellular viability assessed by CCK-8 after 24 hours of $NaIO_3$ treatment (n=6). (C and D) Cells were pretreated with 5 nM MitoQ for 1 hour and then exposed to 12.5 mM $NaIO_3$. (C) Mitochondrial superoxide formation after 2 hours of $NaIO_3$ treatment relative to vehicle-treated cells (n=7) and (D) cellular viability assessed by CCK-8 after 24 hours of $NaIO_3$ treatment (n=4). Mean ± SD, ns = non-significant, * $p < 0.05$.
